# 2116. Efficacy of CD388, a Novel Drug Fc-Conjugate (DFC), is Driven by the Small Molecule Neuraminidase Inhibitor (NAI)

**DOI:** 10.1093/ofid/ofad500.1739

**Published:** 2023-11-27

**Authors:** Simon Döhrmann, James Levin, Elizabeth Abelovski, Amanda Almaguer, Rajvir Grewal, Karin Amundson, Joanne Fortier, Thanh Lam, Thomas P Brady, Allen Borchardt, Jason N Cole, Leslie W Tari

**Affiliations:** Cidara Therapeutics, San Diego, California; Cidara Therapeutics, San Diego, California; Cidara Therapeutics, San Diego, California; Cidara Therapeutics, San Diego, California; Immunology, San Diego, California; Cidara Therapeutics, San Diego, California; Cidara Therapeutics, San Diego, California; Cidara Therapeutics, San Diego, California; Cidara Therapeutics, San Diego, California; Cidara Therapeutics, San Diego, California; Cidara Therapeutics, San Diego, California; Cidara Therapeutics, San Diego, California

## Abstract

**Background:**

Influenza prevention remains a significant public health concern that is still not adequately addressed by vaccines or current therapeutic options. Cidara has developed CD388, a multivalent conjugate of a dimeric NAI with a proprietary hIgG1 Fc domain engineered for extended half-life. CD388 is in clinical development (NCT05285137 and NCT05523089) for the prevention of seasonal influenza A and B. Herein we analyze the contribution of Fc-mediated immune effector function to CD388 efficacy.

**Methods:**

CD388, Fc engineered to extend PK, and closely related Fc modified analogues (immune-active ‘IA’ or immune-silent ‘IS’) were generated. CD388-IA can engage Fc gamma receptors (FcγRs) whereas IS fails to engage FcγRs required to trigger Fc-mediated immune effector functions (e.g. antibody-dependent cellular cytotoxicity). Efficacy studies were conducted in BALB/c WT or Fcer1g^-/-^ mice. The Fcer1g^-/-^ mice are deficient in activating FcγRs thereby excluding contribution of Fc-mediated immunity to efficacy. After lethal challenge with influenza A virus, CD388 or analogues were administered subcutaneously or intramuscularly two hours post-infection. Animals were monitored daily for 14 days or longer for survival (< 20% BW loss). For viral burden quantification, lungs were harvested on day 4 post-infection and viral titers were determined by plaque assay.

**Results:**

CD388-IA and -IS at comparable drug to antibody ratios (DARs) of 4.5±0.5 (used in the clinical candidate) demonstrated comparable protection (Figure 1) and comparable dose-dependent viral burden and cytokine reduction in a lethal mouse model (Table 1). Additionally, the CD388-IA analogue at DAR 4.7 was protective in Fcer1g^-/-^ mice at the same doses required for protection in WT mice. However, a CD388-IA analogue at low DAR of 1, that has reduced antiviral activity, demonstrated improved efficacy in WT mice as compared to KO mice (Figure 2).

**Figure 1.**
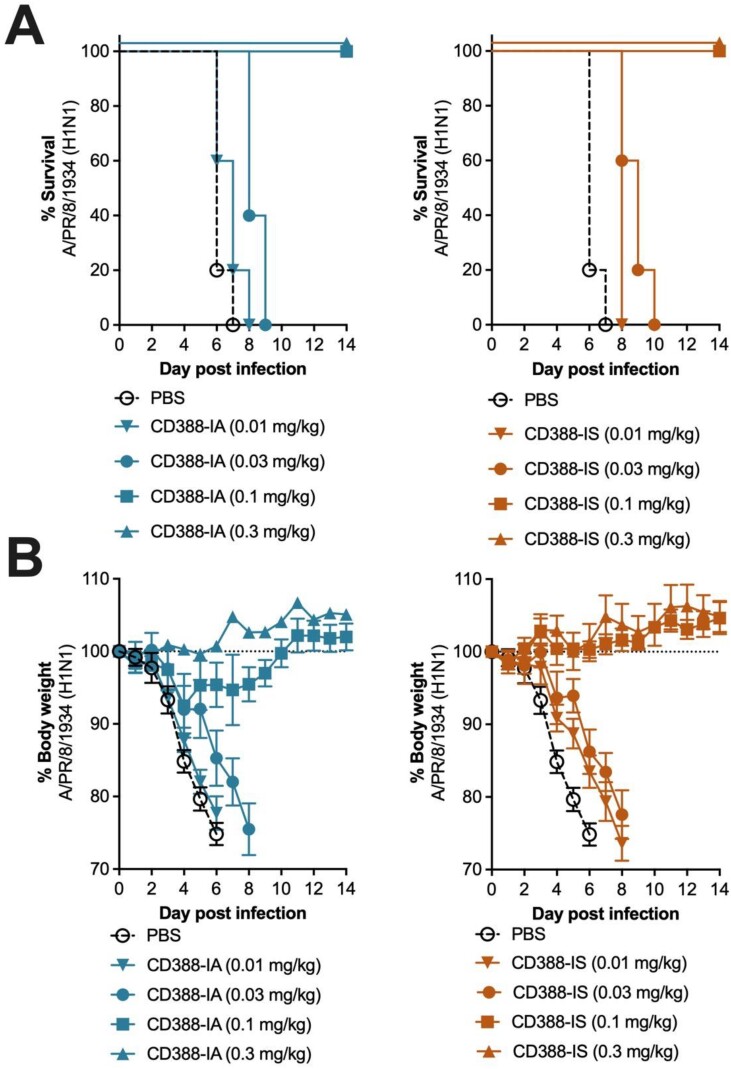
Efficacy of CD388-IA and CD388-IS against A/PR/8/1934 (H1N1) in a lethal mouse model in BALB/c WT mice showing survival (A) and body weight (B)

**Figure 2.**
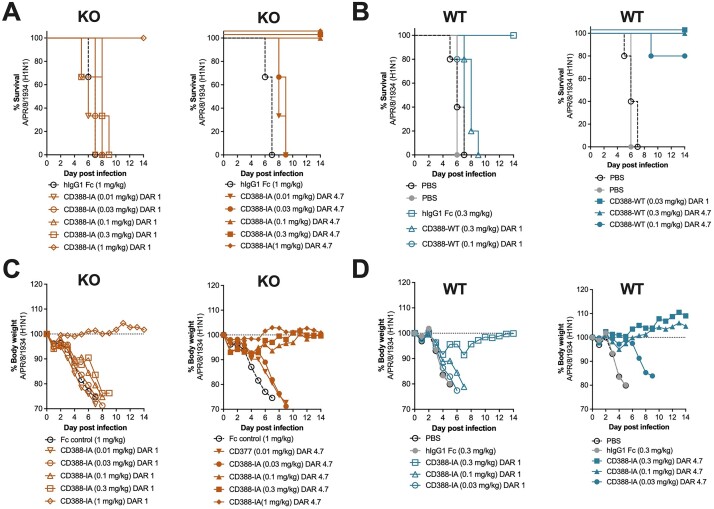
Efficacy of CD388-IA at DAR 1 or 4.7 against A/PR/8/1934 (H1N1) in a lethal mouse model in BALB/c WT (B, D) or KO (A, C) mice

**Table 1.**
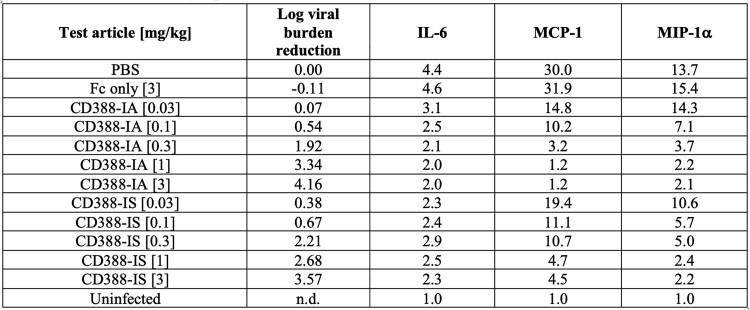
Viral burden reduction and cytokine levels in fold-change versus uninfected control for IL-6, MIP-1□ and MCP-1 on day 4 post-infection in a lethal influenza A/H1N1 mouse model.

**Conclusion:**

These data combined demonstrate that the efficacy of CD388 at DAR 4.5±0.5 is driven predominantly by the intrinsic antiviral activity of the small molecule NAI and is independent of the contribution of Fc-mediated effector functions.

**Disclosures:**

**Simon Döhrmann, PhD**, Cidara Therapeutics: Stocks/Bonds **James Levin, PhD**, Cidara Therapeutics: Stocks/Bonds **Elizabeth Abelovski, B.S.**, Cidara Therapeutics: Ownership Interest **Amanda Almaguer, Bachelors**, Cidara Therapeutics: Stocks/Bonds **Rajvir Grewal, n/a**, Cidara Therapeutics: Ownership Interest **Karin Amundson, BSc**, Cidara Therapeutics: Stocks/Bonds **Joanne Fortier, BSc**, Cidara Therapeutics: Stocks/Bonds **Thanh Lam, PhD**, Cidara Therapeutics: Stocks/Bonds **Thomas P. Brady, Ph.D.**, Cidara Therapeutics: Stocks/Bonds **Allen Borchardt, PhD**, Cidara Therapeutics: Stocks/Bonds **Jason N. Cole, Ph.D.**, Cidara Therapeutics: Stocks/Bonds **Leslie W. Tari, Ph.D.**, Cidara Therapeutics: Stocks/Bonds

